# Construction and experimental verification of user-friendly molecular subtypes mediated by immune-associated genes in hepatocellular carcinoma

**DOI:** 10.3389/fonc.2022.924059

**Published:** 2022-08-05

**Authors:** Juzheng Yuan, Yang Wang, Xudan Wang, Wenjie Zhang, Rui Ding, Shuqiang Yue, Xiao Li

**Affiliations:** ^1^ Xi’an Medical University, Xi’an, China; ^2^ Department of Hepatobiliary Surgery, Xijing Hospital, Fourth Military Medical University, Xi’an, China; ^3^ College of Life Sciences, Northwest University, Xi’an, China

**Keywords:** hepatocellular carcinoma, immunity, prognosis, immunotherapy, PD-L1

## Abstract

Hepatocellular carcinoma (HCC) accounts for third most cancer death globally, and its prognosis continues to be poor even with many novel therapeutic approaches emerging. The advent of immunotherapy seems to offer new hope, but low response rates are an unresolved problem. To gain further knoeledge of the effect of immune-related genes in HCC, we examined the connection between immune-related genes and the immune microenvironment in HCC through the HCC transcriptome dataset. The study also aimed to construct and experimentally validate user-friendly molecular subtypes mediated by immune-related genes in HCC. The immune cell infiltration patterns differs in HCC adjacent non-disease tissues and cancerous tissues. Patients with HCC could be classified into 2 subtypes: subtype A and subtype B. Specifically, subtype A shows characteristics of a hot tumor, in which the infiltration of cells exhibiting antigens and the expression of other crucial factors associated with immune function are higher than in a cold tumor. In addition, we identified Hub genes for the different subtypes and constructed a prognostic prediction model based on six genes (KLRB1, KLF2, S100A9, MSC, ANXA5, and IMPDH1). Further experimental analysis of HCC samples exhibited that the expression levels of KLF2 and ANXA5 were associated with immune cell infiltration and expression of PD-L1 in cancer tissues. Our work suggests that the expression of immune-related genes has crucial effect on the tumor microenvironment and prognosis of HCC patients and may be associated with immunotherapeutic response, which provides new clues for the widespread and effective application of immunotherapy in HCC.

## Introduction

Primary liver cancer has been identified as among the most prevail cancer and contributes to third most cancer death globally (1). In 18 countries worldwide, death induced by liver cancer is the main reason of cancer death cases (1). Primary liver cancer consist of hepatocellular carcinoma (HCC) (taking up 75%-85%); intrahepatic cholangiocarcinoma (10%-15%) as well as other less common types (1). The leading risk factors are chronic infection caused by either Hepatitis B Virus (HBV) or Hepatitis C Virus (HCV), but also exposure to food contaminated with methoxiflotoxin, overweight, excessive alcohol consumption, type II diabetes, and smoking (2). The precaution and medication of liver cancer are mainly based on liver cancer cells. Over the years, the treatment of liver cancer has made rapid progress, and immunotherapy has gained more and more attetion in the clinical researches regarding liver cancer. Immunotherapy is a form of cancer treatment by inciting particular immune responses to inhibit and kill cancer cells, thus reducing the probability of tumor recurrence and metastasis (3). International guidelines suggest that immunotherapy can be an efficient treatment for liver cancer patient that are in advanced stage. Nevertheless, immunortherapy only works in a fraction of cases. The tumor immune microenvironment is an indispensable part of immunotherapy, while the reaction between different immune cells and tumor cells has become a hot spot in the field. Analyzing the tumor immune microenvironment can contribute to the understanding of the occurrence and development of liver cancer (4). A thorough perception of the diverse approaches in which the microenvironment influences the occurrence, prognosis and metastasis of liver cancer will hopefully bring novel strategies for the precaution and medication of liver cancer based on the the changes within the tumor microenvironment and tumor immunotherapy.

Tumor immunity is significantly connected with the tumor microenvironment (TME), which consists of tumor cells, a bevy of immune cells (including mast cells, macrophages, T and B lymphocytes and natural killer cells), and stromal cells (eg. endothelial cells along with stromal cells), and becomes a decisive part in the natural prognosis of tumor (5). The tumor microenvironment of hepatocellular carcinoma is usually seperated into cellular microenvironment and non-cellular microenvironment. The liver regeneration microenvironment invariably contains the cellular components of the pathophysiological changes in the two parts of TME, such as fibroblasts (cancer-associated fibroblasts or CAF), immune cells (B lymphocytes, T lymphocytes, NK cells and natural killer T cells) Cells and TAM) and endothelial cells (EC) become a key part in tumor-matrix interactions that regulate the biological activity of HCC (6). Inflammatory cytokines like IL-6, IL-1, and TNF serve as driving regulators in THE development of HCC (7). IL-6 in HCC (8). Tumor associated macrophages (TAM) is also involved as a promoter of HCC. The high infiltration of the M2 subtype is closly related to the aggressive phenotype of HCC (9) and the number of tumor associated macrophages is related to the number of tumor microvessel in HCC tussues, which indicated that TAM may enhanced tumor angiogenesis (10). In cirrhosis and HCC serum, TNF-α levels was notably higher, indicating that this cytokine is greatly involved in tumor development (11).

To have a deeper understanding of the pathogenesis of HCC, 371 tumor samples were analyzed by RNA-SEQ bioinformatics, and the changes in the tumor immune microenvironment in HCC were further explored. This study also aimed to construct and experimentally validate user-friendly molecular subtypes mediated by immune-related genes in hepatocellular carcinoma. The results indicated that the status of immune cells could reflect the characteristics of tumors, and the invasion of immune cells could conject the prognosis of HCC. The biological function of subtype A was discussed after the new immunotyping. Subtype A showed the characteristics of a hot tumor and had higher immune cell infiltration status. The Hub gene was identified and constructed based on 6 genes (KLRB1, KLF2, S100A9,MSC,ANXA5 and IMPDH1), which will help to for a predictive analysis of the tumor immune microenvironment in HCC patients. Analysis of surgical samples indicated that the expressing levels of KLF2 and ANXA5 were associated with infiltrating immune cells and the expression of PD-L1 at the immune checkpoint in tumor tissues Our analysis showed that immune microenvironment and tumor immunity serve a crucial part in HCC.

## Materials and method

### Datasets and preprocessing

Include RNA-Seq data from HCC samples from the TCGA database ((Level-3 HTseq-FPKM), excluding duplicate sequencing samples from the same patient: TCGA-DD-AACA-02B- 11R-A41C-07, TCGA-DD-AACA-02A-11R-A41C-07, TCGA-ZS-A9CF-02A-11R-A38B-07, 50 normal samples, and 371 tumor samples were finally included for RNA-seq bioinformatics analysis. In addition, excluding the lack of complete follow-up information, patients whose survival time is 0 days, and 365 HCC patients in TCGA were included for survival analysis and model construction. In addition, 231 HCC patients in ICGC, which is known as the International Cancer Genome Consortium database, and the GSE76427 dataset (115 cases) in the GEO database were included as an validation cohort. It is worth mentioning that when conducting validation, we used the sva package to perform background correction, normalization, and expression calculations on the genes involved in modeling to ensure validation comparability.

### Immune infiltration and cluster analysis

In immune cell survival analysis and comparison, the ssGSEA algorithm was applied to estimate the content of 23 immuno cells. In addition, during the verification of immune cell content, immune cells was re-estimated withthe CIBERSORT algorithm, and samples, whose p value is calculated less than 0.05, were included in further analysis. The ESTIMATE algorithm was used to determine the immune score, mesenchymal score, and tumor purity to reflect immune microenvironment status. Consensus clustering was performed on the entire immune cells by the R package ConsensusClusterPlus, and the parameters were set as rep = 1000, pItem = 0.8, pfeature = 1, and the optimal cluster number was caluculated by CDF map as well as heat map.

### Differentially expressed gene selection

Differentially expressed genes analyzed from the TCGA-HCC cohort between cold and hot tumors were calculated with the limma package. Adjusted p-values less than 0.05 and absolute fold changes higher than 0.5 served as cutoffs for the selection of differentially expressed genes.

### RNA extraction and qRT-PCR

Total RNA from frozen tissue was primarily isolated theeough the use of RNAiso Plus reagent (Takara Biotechnology, Dalian, China) as stated by the standard protocol. Reverse transcription was applied by a PrimeScript Master Mix (Takara Biotechnology, Dalian, China),the process was proceeded at 37˚C for 15 min. Then the mRNA expression levels were determined using an SYBR Premix EX TaqTM II (Takara Biotechnology, Dalian, China) on a Bio-Rad IQ5 assay system (Bio-Rad Laboratories, U.S.A.) with the following PCR conditions: pre-denaturation at 95˚C for 30 s, 5sdenaturation at 95˚C, 30 s annealing at 60˚C, and the complete synthesis progress were set for 40 cycles. GAPDH was utilized as an internal reference to normalize the mRNA expressing levels of the target genes. The mRNA expression was determined using the comparative Ct (2^-ΔΔCq^) method. The designed primers were synthesized by TsingKe Biotech (Beijing, China) and the primer sequence was shown in **Table 1**.

**Table 1:** Primer sequence.

Gene name	Primer sequence
KLF2	Forward GGGGTGAGTTCCCCATTCTG
	Reverse CCAATGCACACAACAGGTGG
ANXA5	Forward AGCGGGCTGATGCAGAAAC
	Reverse ACTTCGGGATGTCAACAGAGT
KLRB1	Forward TGGCATCAATTTGCCCTGAAA
	Reverse TCCAAGGGTTGACAGTGTGAG
S100A9	Forward GGTCATAGAACACATCATGGAGG
	Reverse GGCCTGGCTTATGGTGGTG
MSC	Forward CCCCGACACTAAGCTCTCCA
	Reverse GTAGCCGTTCTCATAGCGGT
IMPDH1	Forward CAGCAGGTGTGACGTTGAAAG
	Reverse AGCTCATCGCAATCATTGACG
GAPDH	Forward AGAAGGCTGGGGCTCATTTG
	Reverse AGGGGCCATCCACAGTCTTC

### Protein network analysis for enrichment analysis

Gene Ontology (GO) is used to annotate the molecular functions, biological processes in combination of cellular components of genes. Gene pathways were then annotated using the Kyoto Encyclopedia of Genes and Genomes also known as KEGG. GO and KEGG analyzation were cnoducted using the cluster profile package. P-value less than 0.05 and q-value less than 0.05 were regarded significant enrichment pathways. The STRING database was used to construct the PPI network and further visualized by Cytoscape.

### Immunofluorescence

The 30 paraffin-embedded hepatocellular carcinoma samples were collected from Xijing Hospital. Paraffin sections (5 μm thick) were immersed in xylene I for 15 min, xylene II for 15 minutes, anhydrous ethanol I for 5 min, followed by anhydrous ethanol II for 5 minutes, then 85% alcohol for 5 minutes, as well as 75% alcohol for 5 minutes, and rinsed with distilled water. The tissue sections were then put in a repair box with Ethylene Diamine Tetraacetie Acid antigen repairing buffer (PH8.0) and heated with microwave oven for antigen repair. The microwave oven was set at medium heat for 8 minutes until boiling, cease for 8 minutes, and in turn set for 7 minutes at medium-low heat, during which mind to prevent the buffer from evaporating excessively and the slides should not be dried. After cooling in room temperature, the slides were put in Phosphate Buffered Saline (pH 7.4) and washed 3 times while shaking with a decolorization shaker for 5 minutes every time. Next, after the section was slightly shaken and dried, a circle was drawn surrounding the tissue with the usage of a histochemical pen to prevent the antibody from flowing away. After shaking off the PBS, Body Surface Area was added dropwise to the sections and closed for 30 minutes. After moderately shaking off the blocking solution, add the diluted primary antibody dropwise onto the section, followed by overnight incubation at 4°C in a wet box. (Add a considerate amount of water to the wet box to prevent antibody evaporation). The primary antibodies used in this study were rabbit monoclonal anti-CD45 (1:100, Abcam ab40763, U.S.A.) and rabbit polyclonal PD-L1 (1:1000, Service GB11339, China). The sections were washed 3 times in PBS (pH 7.4) on a decolorized shaker for 5 minutes each time. After slightly shaken and dried, the sections were incubated with Cy3-labeled secondary antibodies (Goat anti-rabbit IgG, 1:300, Service GB21303 or GB25303, China) for 50 minutes at room temperature away from light. The sections were reserved in PBS (pH 7.4) onto a decolorization shaker and washed 3 times for 5 minutes each. The sections were gently shaken and dried, then DAPI dye was dripped dropwise into the circle and reserved for 10 min at room temperature away from light. The sections were placed in PBS (pH 7.4) on a decolorization shaker and washed 3 times for 5 minutes each. Autofluorescence quencher was dripped into the circle and incubated for 5 minutes, and then the sections were rinsed for 10 minutes with running water. The sections were moderately shaken and then sealed by an anti-fluorescence quenching sealer. Finally, the sections were observed with a fluorescent microscope.

### Construction of the predictive model

The least absolute shrinkage and selection operator (LASSO) model was utilized to dismiss high collinearity-related genes to construct a risk model. Risk scoring formulas were developed by combining gene expression values weighted by their LASSO-Cox coefficients. Univariable and Multivariable stepwise regression Cox regression analysis was applied to determine the prognostic value of risk scores across the whole dataset and externally validated datasets. A time-dependent receiver operating characteristic (Troc) curve was applied to compare the predictive accuracy of risk scores with traditional clinicopathological parameters. The survival ROC package was utilized to draw ROC curves and assess the area under the curve (AUC).

### Statistical analysis

The correlation coefficients were determined by Spearman correlation analysis. Kaplan-meier method was applied to plot OS using χ2 test and log-rank test was applied to identify statistical differences. Univariate Cox regression was utilized to calculate risk ratio and multivariate Cox regression was applied to distinguish independent survival factors P<0.05 was regarded statistically significant.

## Result

### Prognostic value of infiltrating immune cells in HCC

First, we calculated the enrichment scores (ES) of 23 immune cells using the ssGSEA algorithm, and merged them with the survival information in a matrix,. The Cox regression analysis method was applied in survival analysis.

**Figure 1:**
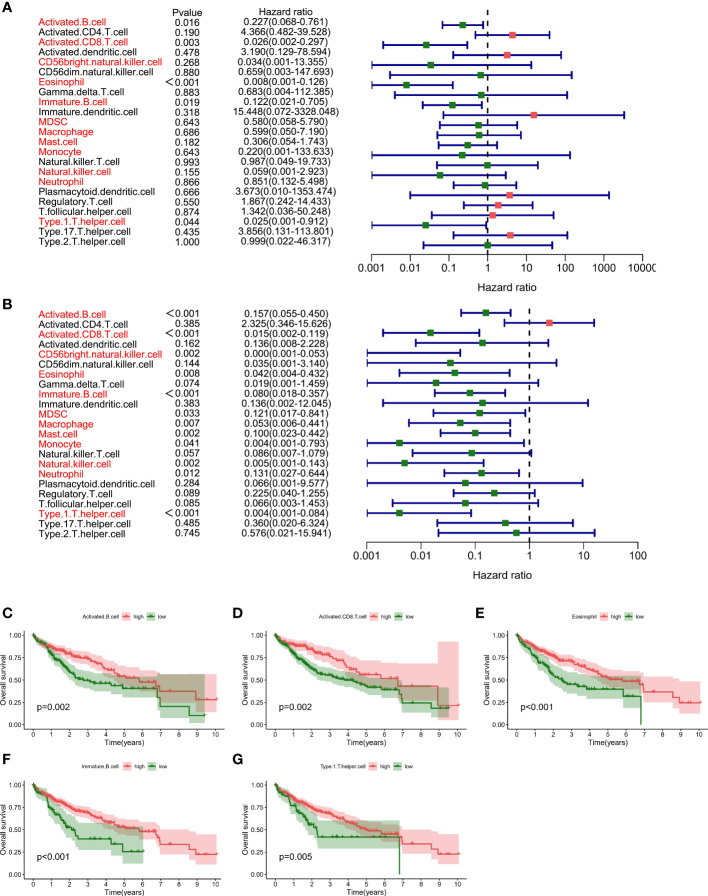
Prognostic value of immune cell infiltration in HCC **(A)**. Survival analysis by Cox regression analysis illustrated the correlation of 23 immuno- cells with overall survival condition **(B)**. Survival analysis using the Cox regression analysis method shows the association of 23 immune cells with progression-free interval (PFI) **(C–G)**. Kaplan-Meier survival analyzation and log-ranking test are performed for the verification regarding the correlation of specific immune cells in survival.

The results showed that in terms of overall survival (OS), massive infiltration of Activated B cells, Immature B cells, Activated CD8 T cells, Eosinophil, as well as Type 1 T helper cells were related to better patient survival (**Figure 1A**). In terms of progression-free interval (PFI), Activated B cell and CD8 T cell, Eosinophil, CD56 bright natural killer cell, Macrophage, Mast cell, Immature B cell, MDSC, Monocyte, Neutrophil, and Type 1 T helper cell can predict better survival outcomes (**Figure 1B**). Additionally, a Kaplan-Meier survival analysis along with a log-rank test on immune cells associated with OS in cox regression were applied, again clarifying their association with survival (**Figures 1C–G**). These data implied that the status of immune cells is able to reflect the characteristics of tumors, and the immune cells infiltration has the function of inferring the prognosis of HCC.

### Different patterns of immuno cell infiltration in HCC adjacent tissues and tumor tissues

**Figure 2:**
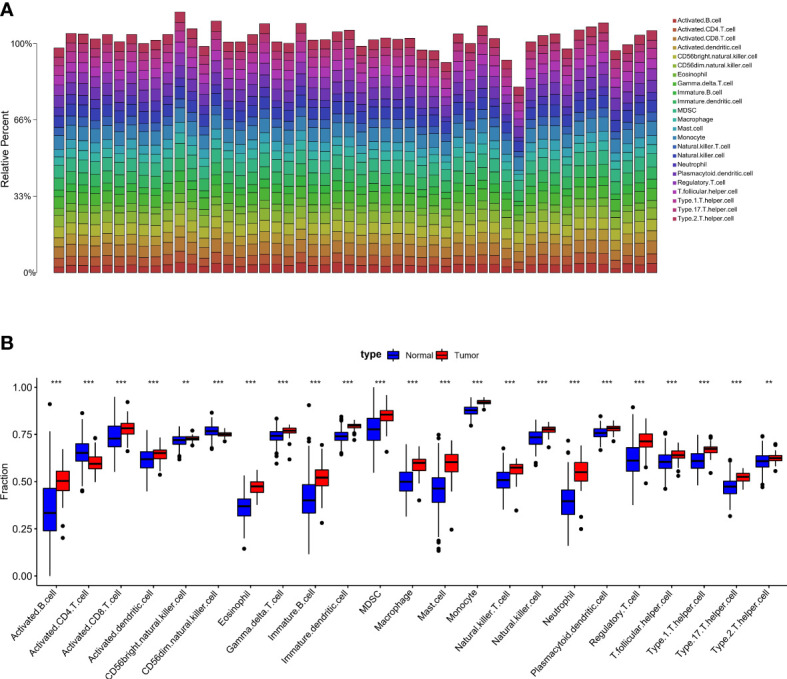
Comparison of immuno-cell infiltration in tumor and adjacent non-tumor sectin **(A)**. Heat map showed the tissue infiltration ratio of 23 immuno-cells in each paracancerous tissue sample **(B)**. Box plot statistics show the difference regarding the infiltration ratio of each immune cell in HCC samples and adjacent tissue samples (**p < .01,***p < .001).

**Figure 3:**
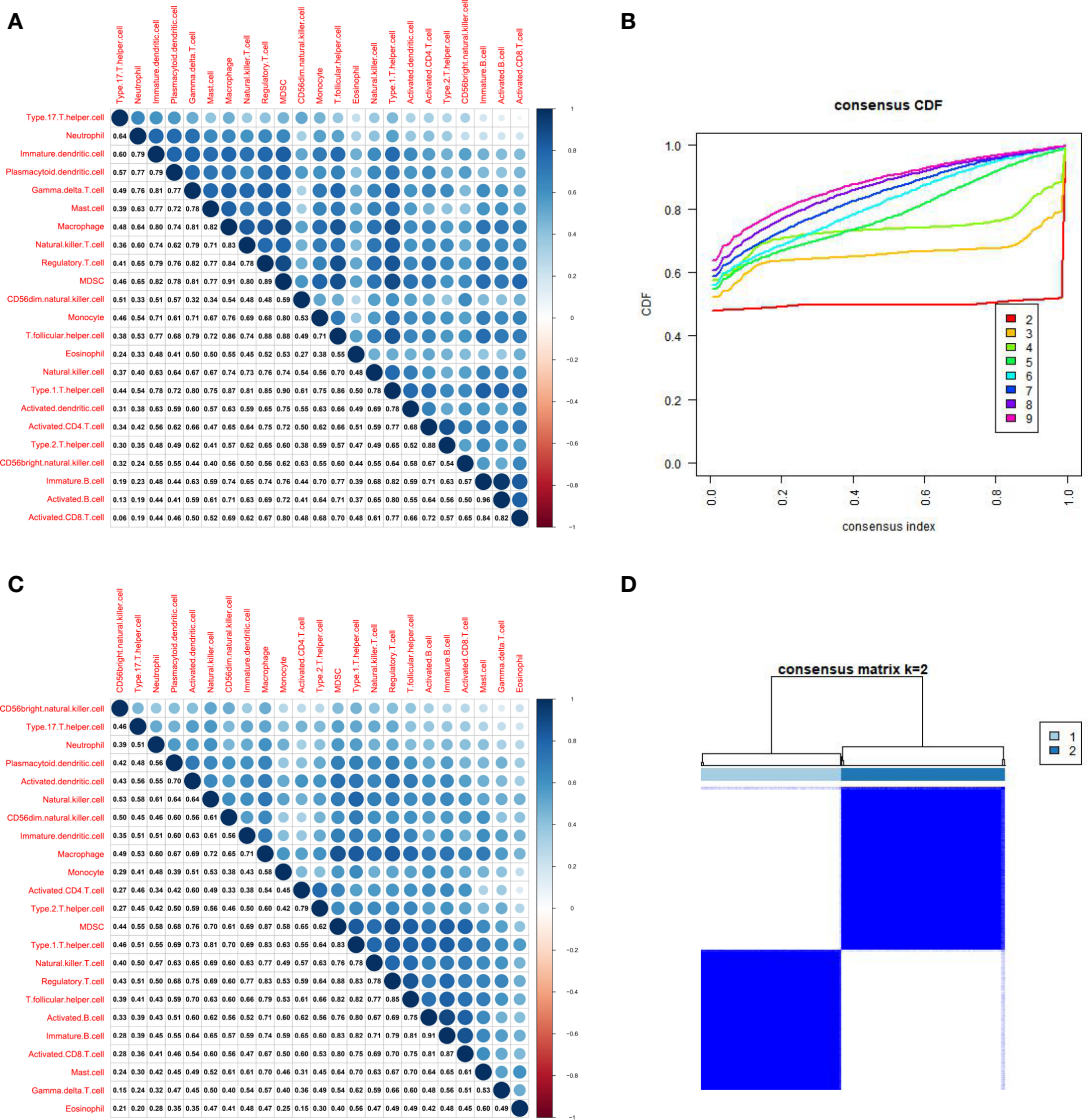
Differentialized immune cell infiltration patterns are present in HCC samples. **(A)**. Correlation analysis of 23 kinds of immune cells based on ssGSEA algorithm shows the correlation of infiltration ratio of each immune cell in paracancerous tissue (The size of the circle represents the size of the correlation). **(B)**. Correlation analysis of 23 kinds of immune cells based on ssGSEA algorithm shows the correlation of infiltration ratio of each immune cell in HCC tissue (The size of the circle represents the size of the correlation). **(C)**. CDF results showed different immune infiltration patterns in HCC samples, according to unsupervised clustering of HCC samples **(D)**. The heat map shows that HCC patients can be divided into 2 subtypes (subtype A and subtype B).

By analyzing the relative distributions of immuno cells in tumor loci and adjoining non-tumor loci in each sample, a marked heterogeneity in HCC and adjacent tumor cells was found (**Figure 2A**, **Supplementary Figure 1**). We then compared the immune cells infiltration in tumor as well as adjacent non-tumor tissues. The findings indicated that most immune cells showed high infiltration levels in HCC tissues, and only CD56 dim natural killer cells, activated CD4 T cells, were up-regulated in paracancerous tissues (**Figure 2B**). Most immune cells respond to tumor neoantigens. In addition, because of tumor-immune interaction appears to be a course involving a series of cell types, it is pivotal to distinguish the relationship between different cells, so correlation analysis was conducted on 23 immune cells utilizing the ssGSEA algorithm. Interestingly, a positive relation between most of the immune cells was found (**Figures 3A, B**). Activated CD8 T cells in normal tissues exhibited the strongest positive association with Immature B cells (r=0.84), and the results in HCC samples were similar, and Activated CD8 T cells also exhibited the most significant positive association with Immature B cells (r=0.87). To better distinguish different immune typing in HCC samples, we performed unsupervised cluster typing on the above HCC samples, CDF results (**Figure 3C**) and heatmap (**Figure 3D**) showed that, according to different immune infiltration patterns, HCC Patients can be divided into 2 subtypes (subtype A and subtype B).

### Establishment of novel immunophenotyping of HCC

**Figure 4:**
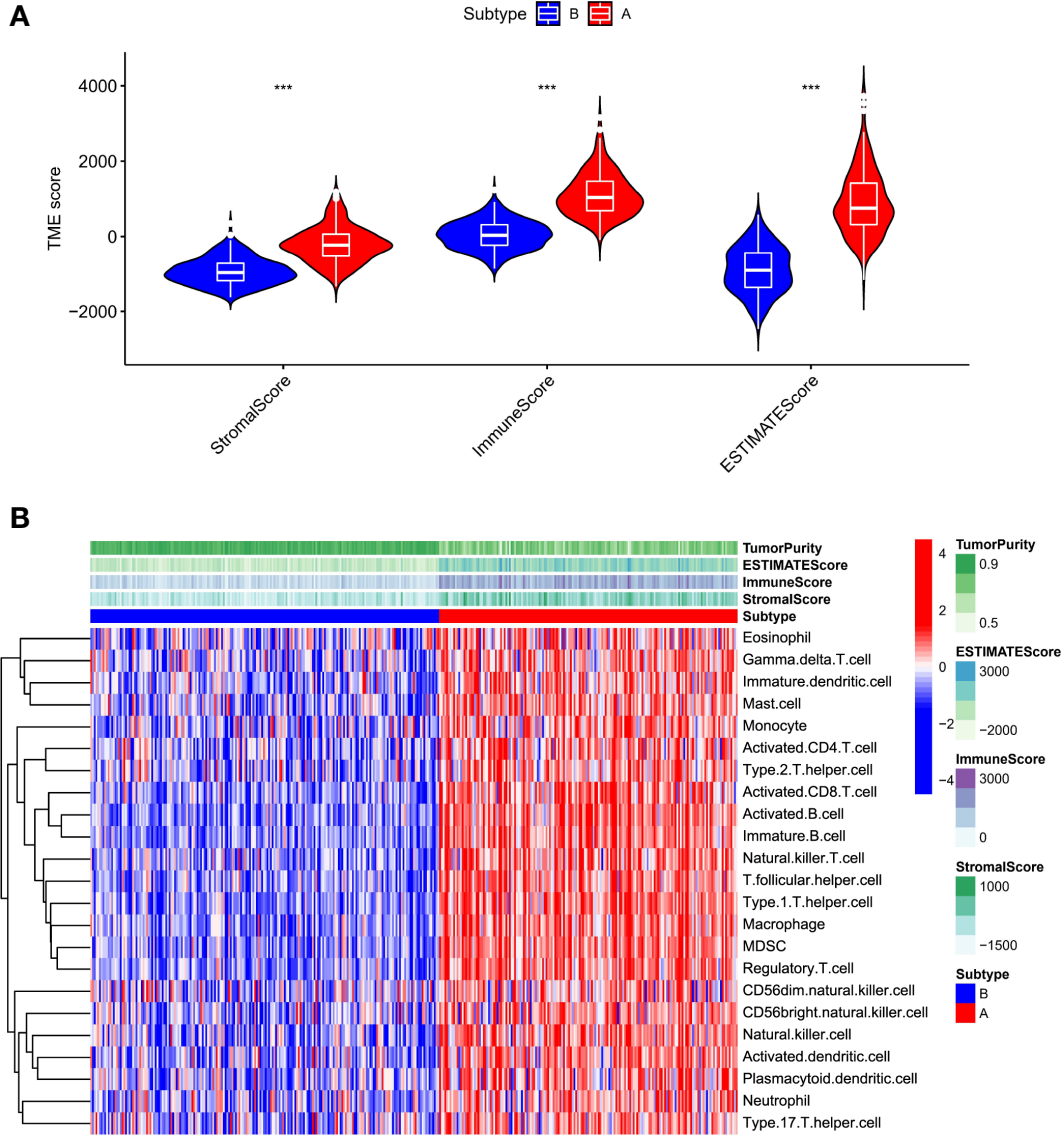
Characterization of subtype A and B HCC samples. **(A)**. The ESTIMATE algorithm calculates the stromal score, immune score, and estimates the score of subtype A and B HCC samples **(B)**. Heatmap illustrated that subtypes A and B have different immune cell infiltration status and tumor purity scores (***p < .001).

The immune score, stromal score, and estimating score were determined with ESTIMATE algorithm to further analyze the A and B subtypes. Interestingly, all types of scores of subtype A dominated (**Figure 4A**).

**Figure 5:**
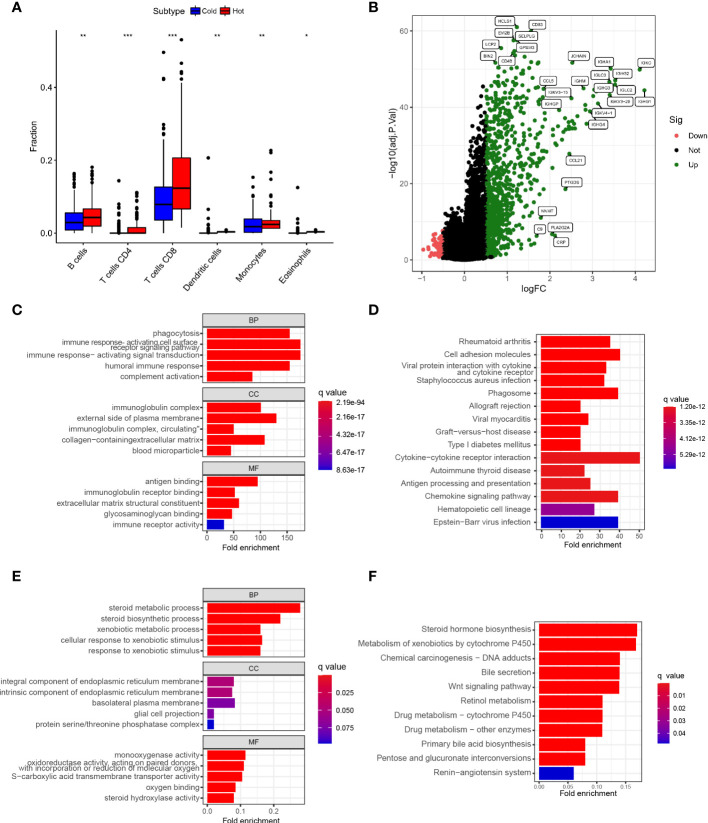
Establishment of Novel Immunophenotyping of HCC **(A)**. Box plot showed the degree of infiltration of antigen-presenting cells,eosinophils and T cellsin subtypes A and B of HCC samples **(B)**. Volcano plot showed identification of immune-related genes of differentially expressed genes (DEGs) between hot and cold tumors **(C)**. Bar plot showed the results of GO enrichment analysis of hot tumor-related genes **(D)**. Bar plot showed the results of KEGG enrichment analysis of hot tumor-related genes **(E)**. Bubble plot showed the results of GO analysis of cold tumor-related genes **(F)**. Bubble plot illustrated the results of KEGG enrichment analysis of cold tumor-related genes (*p < .05, **p < .01, ***p < .001).

Relevant literature suggests that hot tumors imply tumors with higher immune infiltration and tend to profit from immunotherapy, whereas cold tumors with low levels of immune infiltration tend to develop resistance to immunotherapy. Therefore, we made a bold assumption that the subtype A we identified is likely to display the properties of a hot tumor. The heat map combining the results of various immune algorithms showed that subtype A had a higher immune cell infiltration state and a lower tumor purity score (**Figure 4B**). To further test our conjecture, we calculated and iterated 1000 times using the CIBERSORT method on the RNA-seq data to estimate the content of immune cells again. Interestingly, the infiltration of antigen-presenting cells was higher in hot tumors compared with cold tumors, which further confirmed our definition of a novel subtype of HCC (**Figure 5A**). In addition, we downloaded 2483 gene sets (IRGs) that are closely related to immunity from the GeneCard database. In the identification of differentially expressed genes (DEGs) in hot and cold tumors (**Figure 5B**), we found that the vast majority of immune Related genes (295) had a greater expression level in hot tumors (**Supplementary Figure 2A**), and only a few immune-related genes (only 6) were expressed at a higher level in cold tumors (**Supplementary Figure 2B**). Both steps showed a higher degree of infiltration of antigen-presenting cells and other crucial factors associated with immune functions in hot tumors compared to cold tumors, which further confirmed our definition of them. Additionally, we further confirmed higher levels of immune activation in hot tumors using GO Gene Ontology; KEGG-Kyoto Encyclopedia of Genes and Genomesenrichment analysis. We found that cell surfacing receptor signaling pathway capable of stimulating immuno response, immuno response-activated signaling, and complement activation were enriched in hot tumors (**Figures 5C, D** **)**. In cold tumors, the steroid metabolic process and steroid hormone biosynthesis are mainly enriched (**Figures 5E, F**), suggesting that our metabolism-related pathways may be closely related to immune suppression.

### Identify Hub genes and build a predictive model

**Figure 6:**
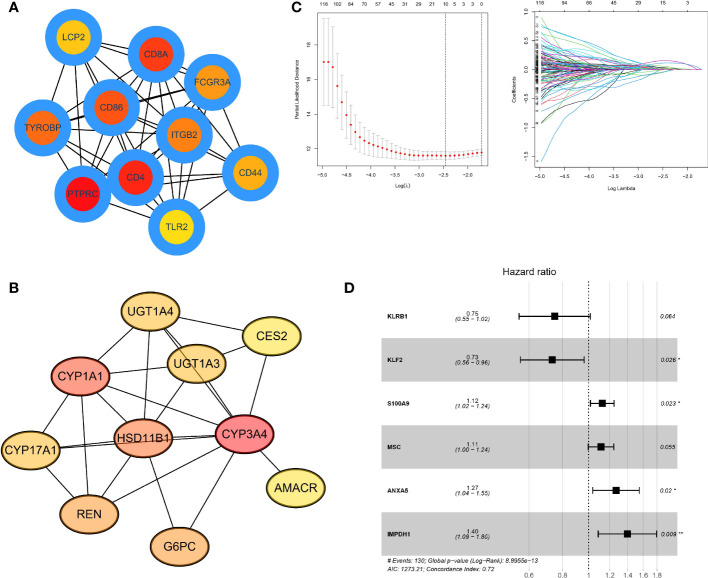
Identifying Hub genes and building predictive models **(A)**. PPI network listed the highest 10 hub genes within hot tumors **(B)**. PPI network listed the highest 10 central genes within cold tumors **(C)**. LASSO-COX regression model to identify genes in the training cohort and determine the prognostic model. The ordinate of the left figure represents the error of the model, and the lower ordinate is log(λ). There are two numerical dotted lines, the left is the line with the lowest error, and the right is the line with fewer features. The ordinate on the right is the value of the coefficient, and the abscissa is log(λ). As the value of λ changes, the later the coefficient is compressed to 0, the more important the variable is. **(D)**. Forest plot showed a gene set of 6 genes predicting survival value. The genes after LASSO regression were analyzed by Cox regression using multivariable step-up regression method (*p < .05, **p < .01).

First, we developed a PPI network in the STRING database and identified hub genes by computational topological rank in Cytoscape software. The top 10 hub genes within hot tumors were LCP2, CD8A, FCGR3A, CD86, ITGB2, CD44, TLR2, CD4, PTPRC, and TYROBP (**Figure 6A**). In cold tumors, the top 10 genes were UGT1A4, CES2, UGT1A3, CYP3A4, AMACR, HSD11B1, G6PC, CYP1A1, CYP17A, and REN (**Figure 6B**). To assess differential gene values in inferring survival condition in HCC, we utilized TCGA as the training cohort with overall survival (OS) as the outcome. A LASSO-COX regression model was then utilized to identify genes in the training cohort (**Figure 6C**), and finally, we found a gene set of 6 genes (**Figure 6D**). We also determined the risk value for every patient as follows: risking score = (-0.2900 × expressing level of KLRB1) + (-0.3111 × expressing level of KLF2) + (0.1176 × expressing level of S100A9) + (0.1074 × expressing level of MSC) + (0.2364 × expressing level of ANXA5) + (0.3339 × expressing level of IMPDH1).

### Validation of the predictive model for overall survival in HCC patients

**Figure 7:**
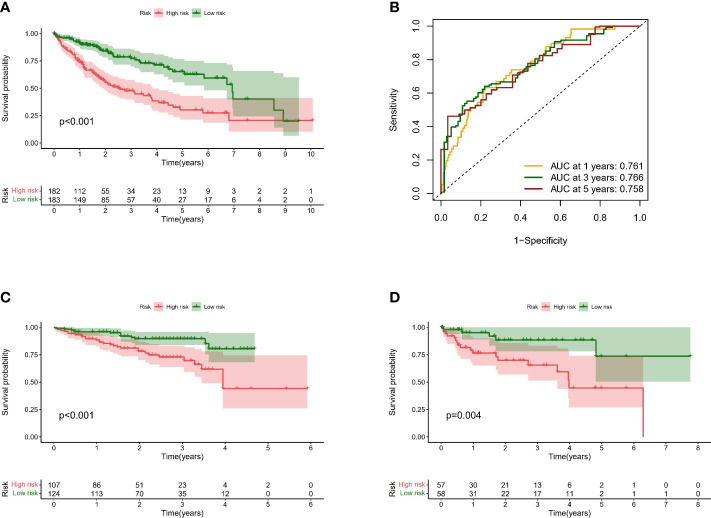
Validation of predictive models for overall survival in HCC patients **(A)**. Line graph showed risk score and possible prediction of patient survival in the TCGA dataset sample **(B)**. Line chart showed the area under the curve for the training cohort at 1, 3, and 5 years showed survival predictions **(C)**. Line graph showed possible predictions of survival for high-risk and low-risk samples in the ICGA dataset sample **(D)**. Line graph showed possible predictions of survival for high-risk and low-risk samples in the GEO dataset sample.

First, to ensure the comparability of validation, we used the sva package to perform background correction, normalization, and expression calculations on the datasets involved in modeling (**Supplementary Figure 2**). We used ICGC and GSE76427 as validation cohorts and calculated the risking score for every patient in the TCGA along with validation cohorts with the above equations, dividing them into high and low-risk groups by the median of the TCGA cohort. Survival analysis indicated that the risk score is able to better distinguish the survival possibility of patients (**Figures 7A, C, D**), and the survival rate of patients with high-risking scores was not satisfactory. The areas under the curve for the training cohort at 1, 3, and 5 years were 0.761, 0.766, and 0.758 (**Figure 7B**). These results suggest that predictive models have great performance in inferring overall survival and have the potential to serve in guiding clinical management.

### Hub gene expressions are associated with immune infiltration and immune checkpoint expression in liver cancer tissue samples

**Figure 8:**
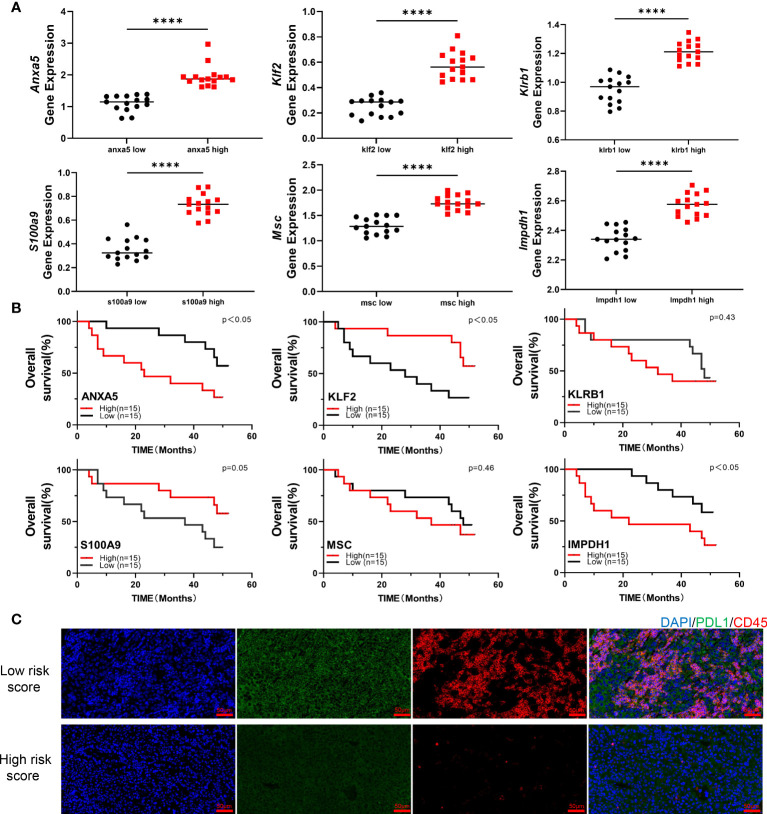
Experimental validation of the prognostic model on the basis of HCC patient samples **(A)**. Dot plots show the expressing level of six prognosis-related genes in HCC patient samples, as measured by qPCR experiments. **(B)**. According to the median expression of qPCR data, the 30 patients were categorized into two groups, and a survival curve was created based on survival time. **(C)**. Immunofluorescence staining showed the expression of CD45 and PDL1 in two groups of HCC tissue samples; the two groups of samples were the specimens with low risk score and the specimens with high risk score, respectively (****p < .0001).

We obtained 30 HCC clinical tissue samples and performed qPCR experiments on these samples to detect the expression levels of 6 hub genes, including KLF2. We separated the samples into low expression groups and high expression groups consistent with the median expression level of each gene in all samples (**Figure 8A**). The outcome of the survival curve indicated that the high expression of ANXA5 and IMPDH1 might be related to a low survical rate and short survival time, while the high expression of KLF2 and S100A9 might be related to a high survical and long survival time (**Figure 8B**). To further confirm the accuracy of this prognostic model discussed in this study, we determined the risk score of 30 patients (**Supplementary Table 1**).Then the 30 samples were divided into the high-risk and low-risk scores groups based on the risk scores,and draw a KM curve between survival time and risk score of these patients (**Supplementary Figure 4**). Next, we performed immunofluorescence staining for CD45 and PDL1 to describe the immune infiltration and differential expression of immune checkpoints in tumor tissues (**Figure 8C**). The results indicated that the samples with low-risk scores had higher immune cell infiltration and PDL1 expression, which proved that our prognostic model is valid and accurate.

## Discussion

HCC is a prevailing liver malignant tumor globally, with highest incident in East Asia (17.8 cases per 100,000 persons), posing a grave risk to public health (12, 13). Hepatocellular carcinoma is an inflammatory tumor that often emerges gradually in chronic inflammation and is determined by a strong association between tumor cells and the tumor microenvironment, a persistent inflammatory environment that contributes to the transformation of epithelial cells Fibroblasts related to cancer and the establishment of the immunosuppression of myeloid cells pathological conditions (14), accompanied by a variety of immune inhibitory signals, therefore, to understand the immune microenvironment of hepatocellular carcinoma (HCC), for inspection of some immune checkpoint and tags to enhance antitumor effect is very important for liver cancer immunotherapy immune modulators of interferon-alpha Pd-1 (programmed death receptor 1) is expressed in activated T cells, B cells and myeloid cells, and PD-L1 and PD-L2 are both expressed in antigen-presenting cells as ligands of PD-1 The binding of PD-1 and PD-L1 can mediate the co-inhibitory signal of T cell activation and down-regulate the stimulation and proliferation of T cells. Hence, the study of PD-1/PD-L1 as a target against tumors is of great significance (15). Among 240 patients suffering primary liver cancer, the prognosis and overall survival rate of patients with high PD-L1 expression are significantly less than those with low or negative PD-L1 expression, and the researchers purposed that the expression level of PD-L1 in patients with liver cancer maybe an independent factor to predict postoperative liver cancer recurrence (16).

The TME regards to the cellular conditions where tumor cells are located, and consists of a amalgamation of tumor stromal cells, soluble molecules, and immune cells. The interaction between tumor cells and their microenvironment plays a major role in tumor development as well as normal tissue growth (17, 18). This paper firstly evaluated the prognostic value of immune cell infiltration in HCC. The results indicated that Activated B cells, Activated CD8 T cells, Eosinophils, Immature B cells, and Type 1 T helper cells were related to better OS and PFI in patients, and could also predict better survival outcomes. The data suggest that the state of immune cells can reflect the characteristics of tumors, and the infiltration of immune cells exhibits the function of infering the prognosis of HCC. The cellular immune response is one of the anti-tumor immune responses, which is mainly dominated by T lymphocyte subsets. Clinical studies have shown (19): when the body’s immune function is enhanced, the proportion of CD8+ T lymphocytes in immune cell subsets increases significantly; while CD8+ T lymphocytes are effector T lymphocytes, which are involved in the body’s immune response.During this process, it plays the role of killing antigens, including cancer antigens, which can reflect the cellular immune status of the body (20, 21). It has been proved in liver cancer that the number of CD8+ T cells infiltrated in tumor tissue is positively correlated with prognosis, that is, the more CD8+ T cells infiltrated in liver cancer tissue, the lower the recurrence rate of patients and the better the prognosis (22).

Next, we analyzed the differentialized immune cell infiltration patterns in HCC adjacent non-tumor section and tumor loci. HCC patients can be divided into 2 subtypes (subtype A and subtype B). Specifically, subtype A showed a characteristic of hot tumors is that compared with cold tumors, hot tumors have a higher degree of infiltration of antigen-presenting cells and other crucial factors associated with immune functions. While traditional cold and hot tumors are usually judged as post-clinical responses, the new subtypes (subtype A and subtype B) we established can be distinguished by cluster analysis, in particular, gene expression levels and coefficients can be identified. KEGG analysis showed that immune response-activated cell surface receptor signaling pathway, immune response-activated signal, and complement activation were enriched in hot tumors; in cold tumors, they were mainly enriched in steroid metabolic process and steroid hormone biosynthesis. Jennifer L et al. found that chronic inflammation promotes the development of HCC (23), while complement activation impairs the process of tumor elimination by the immune system (24). Therefore, insufficient complement administration in the liver may lead to liver cancer through multiple mechanisms. Finally, Hub genes were identified and predictive models were constructed, including the following genes: KLRB1, KLF2, S100A9, MSC, ANXA5, and IMPDH1. Evidence were found suggesting that that KLF2, part of the Krüppel-like factor (KLF) family, is involved in the development of preventing hepatocellular carcinoma (HCC). Yining Li discovered that TGF-β stimulated the KLF2 gene expression in multiple HCC cells. KLF2 was stably expressed in HCCLM3 cells and thus reduced TGF-β-induced wound healing and cancer cell mobility by intervening TGF-β-stimulated promotion of MMP2. The results of this study showed that KLF2 protein exerts tumor suppressor ability in HCC by the negative feedback pathway of TGF-β signaling (25). A calcium-dependent phospholipid-binding annexin- annexin A5 (ANXA5) is associated with the generation, metastasis and development of various cancers. Current studies have shown that in HCC patient tissues, overexpression of ANXA5 is associated in a positive way with the up-regulation of CRKI/II and RAC1, thus promoting the clinical progression and lymphatic metastasis of HCC (26, 27). *In vitro* experiments showed that stable knockout of ANXA5 reduced the proliferation, migration, invasion, and lymph node adhesion of hepatoma Hca-P cells by inhibiting CD34 and VEGF3 increased the adhesion behavior between cells, and reduced tumorigenicity and malignant tumorigenesis in mice (28). KLRB1 encodes CD161 protein, which is expressed in NK cells and T cell subsets, and mainly affects the occurrence and progression of tumors by regulating the cytotoxicity of NK cells. KLRB1 was attenuated in 13 cancers and promoted in kidney cancer. Patients with high KLRB1 expression have more promising prognosis in most cancers, and its expression level is mainly associated with TMB, MSI, and several immune signals of tumors. The expression of KLRB1 is able to influence tumor immune cell infiltration and chemotherapeutic drug sensitivity (29). S100A9 is a member of the S100 family of calcium-binding proteins and is overexpressed in many human tumors in addition to hepatocellular carcinoma (HCC). Studies have showed that S100A9 treatment enhanced the survival and invasiveness of three hepatoma cell lines, HepG2, SMMC-7721, and Huh7, and S100A9 also facilitated tumor development *in vivo* in a xenograft mouse model. S100A9 significantly increased p-p38 and p-ERK1/2 levels, and inhibition of p-p38 and p-ERK1/2 blocked the increase of infiltration and viability caused by S100A9, respectively. Moreover, RAGE-blocking antibody treatment also revked p38 and ERK1/2 activation caused by s00a9, indicating that MAPK activation caused by s100a9 is redulated through RAGE ligation. This result suggests that S100A9 binds to RAGE and activates a RAGE-dependent MAPK signaling cascade, thereby increasing cell growing and invasing in HCC (30). Multipotent mesenchymal stromal cells (MSCs) are capable of being recruited into the HCC microenvironment, become an integral part of the HCC immune microenvironment, and is able to affect tumor development. The function of MSCs in the tumor microenvironment is linked to tumor type as well as immune status. In the tumor immunosuppressive microenvironment, mesenchymal stem cells mainly increase tumor development *via* inhibiting the anti-tumor immunity of the body’s normal immune activity, while serving a part in inhibiting tumor progression. MSCs play a dual part in HCC progression by inducing apoptosis and inhibiting HCC cell amplification, infiltration and invasion *in vitro* and tumor growth and metastasis *in vivo* (31). Inosine monophosphate dehydrogenase (IMPDH) is a crucial enzyme in the biosynthesis of purine nucleotides, and there are mainly two isoforms of IMPDH1 and IMPDH2. The results demonstrated that the expression of IMPDH1 was up-regulated in HCC tissues comparing to adjoining liver tissues, and the higher the level of IMPDH1 expression, the higher the cumulative survival rate of patients. Furthermore, depletion of IMPDH1 in HCC cells inhibited the capability to formulate single-cell colonies *in vitro* and reduced tumor initiation and growth efficiency in immunodeficient mice. Genome-wide transcriptome analysis revealed that loss of IMPDH1 resulted in marked alterations in signaling pathways. This result suggests that IMPDH1 maintains the growth and progression of HCC (32).

In conclusion, this article systematically explored the association between immunotherapy and the immune microenvironment in HCC. Through our study, it was confirmed that genes related to immune infiltration have the function of predicting the prognosis of HCC, and HCC can be divided into two subtypes by immune infiltration-related genes, subtype A and subtype B, corresponding to hot tumors and cold tumors, respectively. And a higher degree of infiltration of cells presenting antigen and other important immune-related factors was found within hot tumors. In addition, we identified the Hub gene and developed a prognostic prediction model based on 6 genes (KLRB1, KLF2, S100A9, MSC, ANXA5, and IMPDH1), KLF2 and ANXA5 were the main related genes. Experimental analysis of surgical samples from HCC patients indicafed that the risk score were associated with immuno cell infiltration and the expression of immune checkpoint PD-L1 in tumor tissues. Finally, Construction and experimental verification of user-friendly molecular subtypes mediated by immune-associated genes in Hepatocellular carcinoma. These findings may provide clues for immunotherapy strategies for HCC.

## Data availability statement

The datasets presented in this study can be found in online repositories. The names of the repository/repositories and accession number(s) can be found in the article/**Supplementary Material**.

## Ethics statement

This study was approved by the Institutional Ethics Committee of First Affiliated Hospital of Fourth Military Medical University and written informed consent was obtained from each participant.

## Author contributions

Conceptualization, XL, JY, YW, XW, WZ,RD and SY. Clinical data collection and collation, YW, CY, JY, and SY. Clinical data analysis, JY and YW. Bioinformatics analysis, JY. Resources, YW and XW. Supervision, SY,XL. Writing-original draft, XL, JY and SY. Writing-review and editing, XL, JY and SY. All authors read and approved the final manuscript.

## Funding

This work was supported by National Natural Science Foundation of China, No. 81672753.

## Conflict of interest

The authors declare that the research was conducted in the absence of any commercial or financial relationships that could be construed as a potential conflict of interest.

## Publisher’s note

All claims expressed in this article are solely those of the authors and do not necessarily represent those of their affiliated organizations, or those of the publisher, the editors and the reviewers. Any product that may be evaluated in this article, or claim that may be made by its manufacturer, is not guaranteed or endorsed by the publisher.

## Supplementary material

Supplementary Figure 1 Comparison of immune cell infiltration in tumor section Heat map showed the tissue infiltration ratio of 23 types of immuno cells in each HCC sample

Supplementary Figure 2 Expression levels of immune-related genes in cold and hot tumors **(A)**. Venn diagram showed the number of immune-related genes that are highly expressed in hot tumors **(B)**. Venn diagram showed the number of highly expressed immune-related genes in cold tumors

Supplementary Figure 3 Background correction, normalization for different datasets **(A)**. PCA analysis showed sample differences before background correction of the dataset **(B)**. PCA analysis showed sample normalization after dataset background correction

Supplementary Figure 4 KM curve between survival time and risk score of 30 HCC patients.
