# Supplementary material for: Construction and experimental verification of user-friendly molecular subtypes mediated by immune-associated genes in hepatocellular carcinoma
Source: Front Oncol. 2022 Aug 5;12:924059. doi: 10.3389/fonc.2022.924059 (PMC9391001; doi:10.3389/fonc.2022.924059)
Supplement: Supplementary file 1 [file Table_1.docx]

| ID | futime | fustat | risk score |
| --- | --- | --- | --- |
| 1 | 45 | 0 | 0.938265172 |
| 2 | 32 | 1 | 1.281701893 |
| 3 | 4 | 1 | 1.116701145 |
| 4 | 51 | 0 | 0.909869048 |
| 5 | 37 | 1 | 1.081178954 |
| 6 | 48 | 0 | 1.093722779 |
| 7 | 49 | 0 | 1.103768821 |
| 8 | 50 | 0 | 1.098023777 |
| 9 | 7 | 1 | 1.278630764 |
| 10 | 23 | 1 | 1.181702804 |
| 11 | 43 | 1 | 1.372913893 |
| 12 | 44 | 1 | 0.848213562 |
| 13 | 9 | 1 | 1.359542283 |
| 14 | 47 | 1 | 1.038784292 |
| 15 | 28 | 1 | 1.057722676 |
| 16 | 7 | 1 | 1.371876689 |
| 17 | 50 | 0 | 1.533967009 |
| 18 | 49 | 0 | 1.322590611 |
| 19 | 48 | 1 | 1.076580064 |
| 20 | 49 | 0 | 1.022001373 |
| 21 | 47 | 1 | 1.417715052 |
| 22 | 52 | 0 | 1.062454262 |
| 23 | 22 | 1 | 1.195470826 |
| 24 | 47 | 0 | 1.274803435 |
| 25 | 16 | 1 | 1.283534944 |
| 26 | 50 | 0 | 0.907499248 |
| 27 | 48 | 0 | 1.281197866 |
| 28 | 10 | 1 | 1.047093133 |
| 29 | 44 | 0 | 0.994117474 |
| 30 | 5 | 1 | 1.254237761 |

Supplementary Table 1
