# Supplementary figures and images for: Construction and experimental verification of user-friendly molecular subtypes mediated by immune-associated genes in hepatocellular carcinoma
Source: Front Oncol. 2022 Aug 5;12:924059. doi: 10.3389/fonc.2022.924059 (PMC9391001; doi:10.3389/fonc.2022.924059)

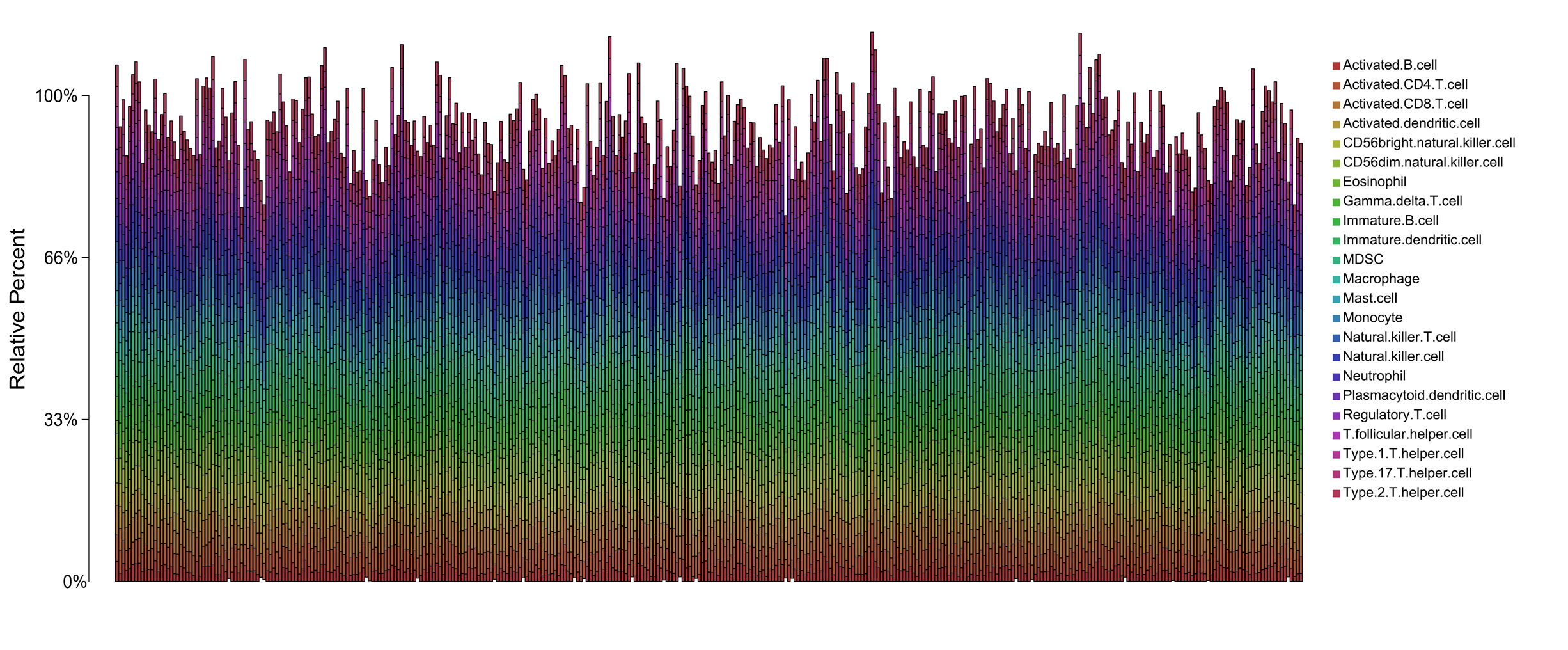

Supplement: Supplementary Figure 1 — Comparison of immune cell infiltration in tumor section Heat map showed the tissue infiltration ratio of 23 types of immuno cells in each HCC sample [file Image_1.tif]

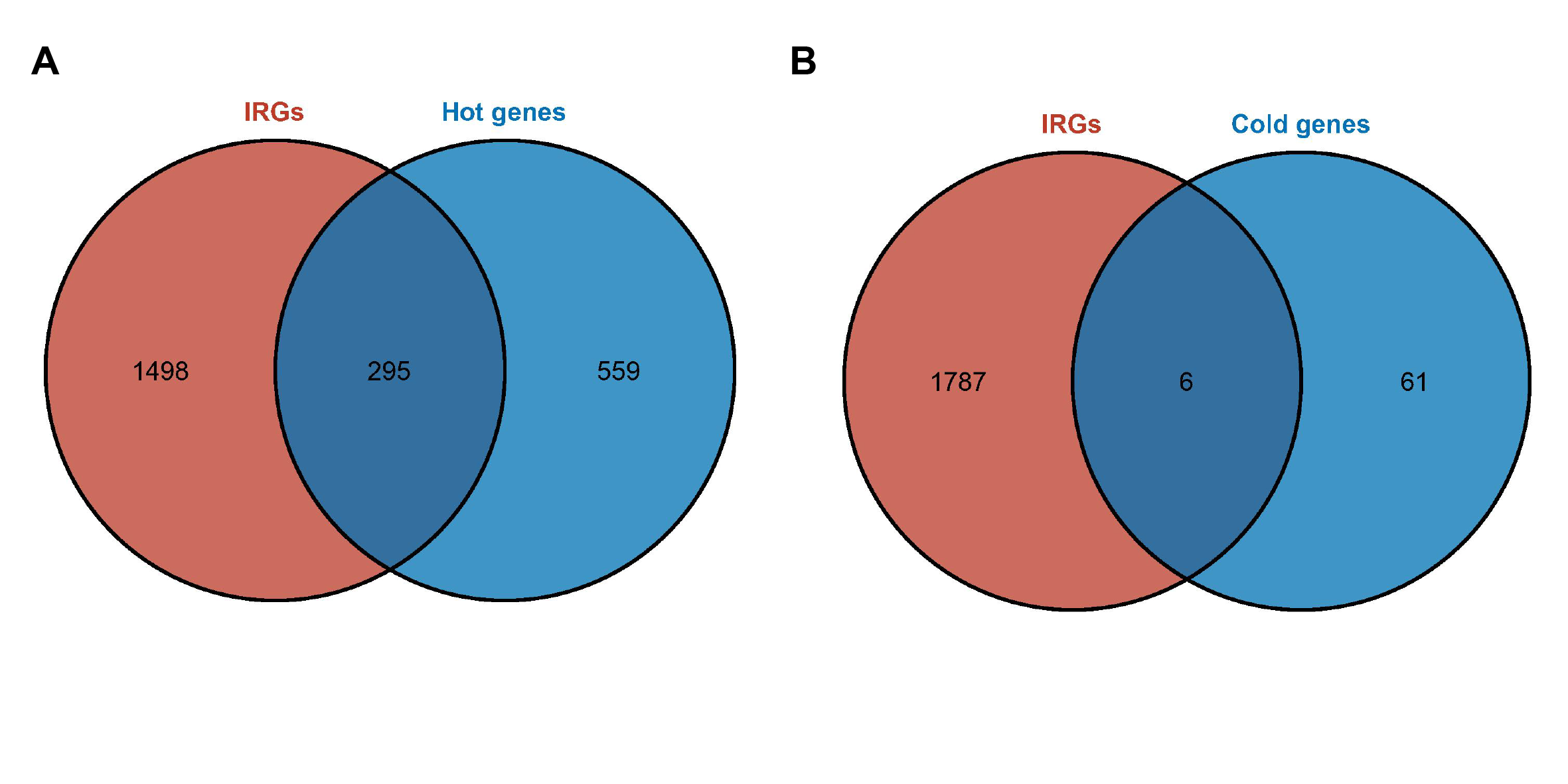

Supplement: Supplementary Figure 2 — Expression levels of immune-related genes in cold and hot tumors (A). Venn diagram showed the number of immune-related genes that are highly expressed in hot tumors (B). Venn diagram showed the number of highly expressed immune-related genes in cold tumors [file Image_2.tif]

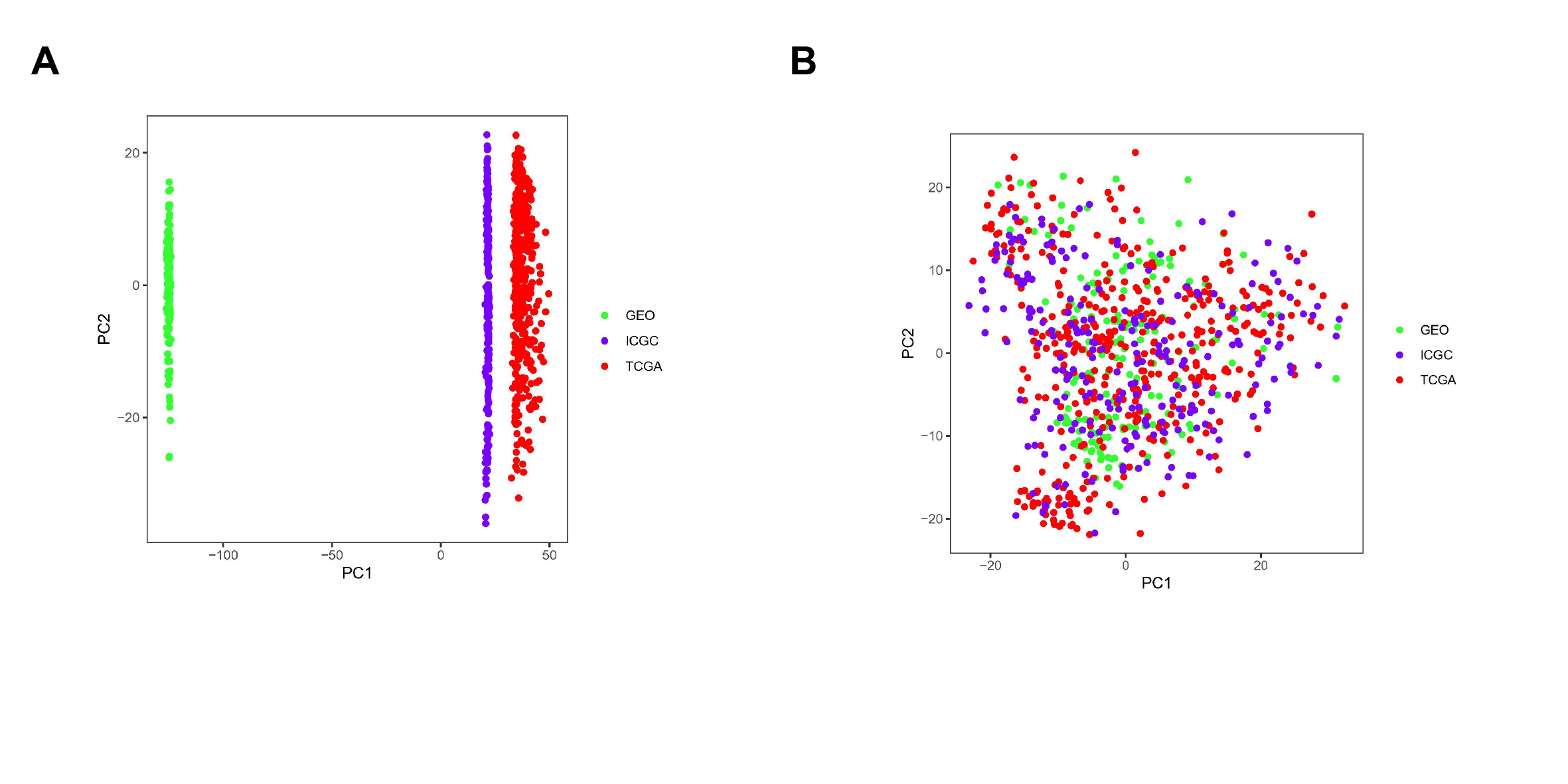

Supplement: Supplementary Figure 3 — Background correction, normalization for different datasets (A). PCA analysis showed sample differences before background correction of the dataset (B). PCA analysis showed sample normalization after dataset background correction [file Image_3.tif]

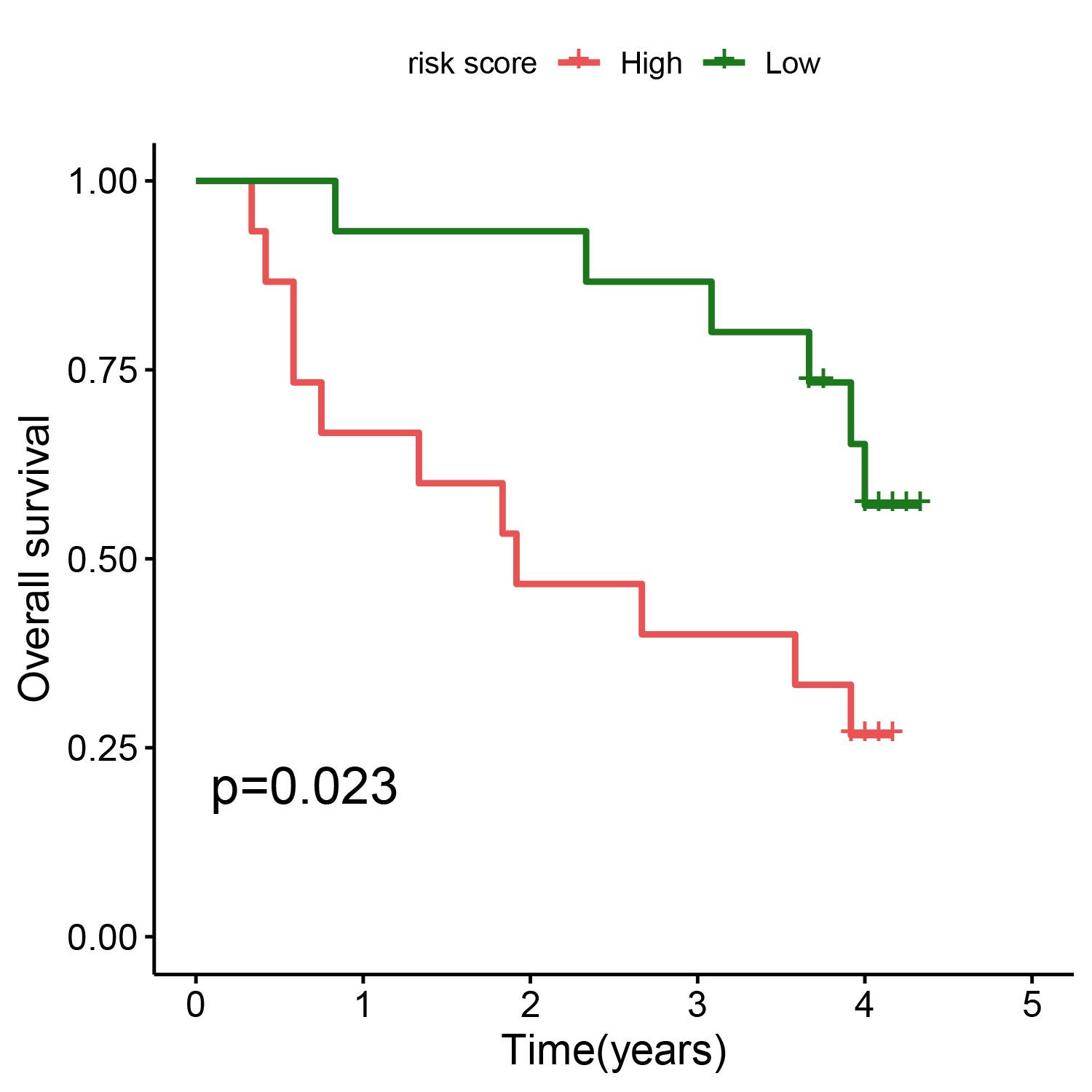

Supplement: Supplementary Figure 4 — KM curve between survival time and risk score of 30 HCC patients. [file Image_4.tif]
